# Development and external validation of a new PTA assessment scale

**DOI:** 10.1186/1471-2377-12-69

**Published:** 2012-08-08

**Authors:** Bram Jacobs, Janneke van Ekert, Lotje PL Vernooy, Peter Dieperink, Teuntje MJC Andriessen, Marc PH Hendriks, Arie B van Vugt, Marjolein AA Emons, George F Borm, Pieter E Vos

**Affiliations:** 1 Department of Neurology, Radboud University Nijmegen Medical Centre, Nijmegen, the Netherlands; 2 Donders Institute for Brain, Cognition and Behaviour, Radboud University Nijmegen, Nijmegen, the Netherlands; 3 Department of Behavioural Sciences, Epilepsy Centre Kempenhaeghe, Heeze, the Netherlands; 4 Department of Emergency Medicine, Radboud University Nijmegen Medical Centre, Nijmegen, the Netherlands; 5 Department of Epidemiology, Biostatistics and HTA, Radboud University Nijmegen Medical Centre, Nijmegen, the Netherlands

**Keywords:** Traumatic brain injury (TBI), Head injury, Post-traumatic amnesia (PTA), PTA assessment scale

## Abstract

**Background:**

Post-traumatic amnesia (PTA) is a key symptom of traumatic brain injury (TBI). Accurate assessment of PTA is imperative in guiding clinical decision making. Our aim was to develop and externally validate a short, examiner independent and practical PTA scale, by selecting the most discriminative items from existing scales and using a three-word memory test.

**Methods:**

Mild, moderate and severe TBI patients and control subjects were assessed in two separate cohorts, one for derivation and one for validation, using a questionnaire comprised of items from existing PTA scales. We tested which individual items best discriminated between TBI patients and controls, represented by sensitivity and specificity. We then created our PTA scale based on these results. This new scale was externally evaluated for its discriminative value using Receiver Operating Characteristic (ROC) analysis and compared to existing PTA scales.

**Results:**

The derivation cohort included 126 TBI patients and 31 control subjects; the validation cohort consisted of 132 patients and 30 controls. A set of seven items was eventually selected to comprise the new PTA scale: age, name of hospital, time, day of week, month, mode of transport and recall of three words. This scale demonstrated adequate discriminative values compared to existing PTA scales on three consecutive administrations in the validation cohort.

**Conclusion:**

We introduce a valid, practical and examiner independent PTA scale, which is suitable for mild TBI patients at the emergency department and yet still valuable for the follow-up of more severely injured TBI patients.

## Background

Post-traumatic amnesia (PTA) is an essential aspect of traumatic brain injury (TBI), characterized by confusion, disorientation, retrograde and anterograde amnesia [1-3]. PTA duration designates injury severity [4,5], and predicts cognitive recovery [6,7], functional outcome [8-11] and return to work [9-13]. Additionally, after mild TBI, the presence and duration of PTA are associated with the risk for intracranial traumatic lesions [5,14-16]. Evaluation of PTA is further used to monitor TBI recovery and to guide therapy and rehabilitation decisions. Despite the importance of accurate PTA assessment, no gold standard for PTA assessment exists, and controversy remains regarding the preferred method to objectively measure the presence and duration of PTA.

Existing PTA scales including the Galveston Orientation and Amnesia Test (GOAT) [17], the (Modified) Oxford PTA Scale (MOPTAS) [18], the Westmead PTA Scale (WPTAS) [19] and the Revised-WPTAS (2004, Ponsford version) [20] use standardized assessment formats (Table 1). Limitations associated with these scales refer to imperfect accuracy, because not all answers to memory questions can be verified. Furthermore, several test items are retrospective in nature [1], and pictures are used instead of words as memory items, which may be impractical, especially in emergency department (ED) settings [21,22]. In addition, test items that require remembering the examiner’s name and face are unfeasible given the fact that longitudinal PTA assessment often requires multiple testing [20]. The item *name of examiner* is often failed by a substantial proportion of control subjects as well [20]. A final area of concern is the level of task difficulty. Although widely accepted as a legitimate test item, the three-picture memory test has been shown to be less sensitive to test PTA than a three-word memory test [22-24].

**Table 1:** The combined PTA questionnaire administered to participants; a composite of items from existing PTA scales

** *Questionnaire* **	** *GOAT* **	** *WPTAS* **	** *R-WPTAS* **	** *MOPTAS* **
** *Reference* **	[17]	[19]	[20]	[18]
01. *Name*	√			
02. *Age*		√	√	√
03. *Date of birth*	√	√	√	√
04. *Residence*	√			
05. *Marital status*				√
06. *Children*				√
07. *Occupation*				√
08. *Recognition face examiner*		√	√	√
09. *Recall name examiner*		√		√
10. *Kind of place*	√			√
11. *Name of place*		√	√	√
12. *City of hospital*	√			
13. *Date of admittance*	√			
14. *Mode of transport*	√			
15. *Last memory preceding injury*	√			√
16. *First memory following injury*	√			√
17. *Period of day*		√	√	
18. *Time of day*	√			
19. *Day of week*	√	√		√
20. *Date*	√			
21. *Month of year*	√	√	√	√
22. *Year*	√	√	√	√
*23. 3-item memory test*		√	√	√
*Maximum Score*	100	12	10	15
*Criterion – PTA present (disease positive)*	*≤ 75*	*≤ 11*	*≤ 9*	*≤ 14*

PTA = post-traumatic amnesia; GOAT = Galveston Orientation and Amnesia Test; WPTAS = Westmead PTA scale; R-WPTAS = revised Westmead PTA scale; MOPTAS = modified Oxford PTA scale.

To overcome the accuracy and practical shortcomings related to existing PTA scales the present study was undertaken. In the first part we constructed an examiner independent PTA scale composed of a set of individual items with the highest discriminative value taken from existing PTA scales. In part two this newly composed scale was subsequently validated in patients and controls, and the concurrent validity was assessed by comparing it to the existing GOAT [17], MOPTAS [18] and (Revised-)WPTAS [19,20] scales.

## Methods

### Subjects

This single center prospective cohort study was executed at the ED and neurological and surgical wards of the Radboud University Nijmegen Medical Centre, a level I trauma centre; the first part of the study (derivation) between 2005 and 2006 and the second part (validation) in 2009.

All consecutive TBI patients, over 16 years of age and admitted to the ED, were eligible for inclusion. TBI was classified, based on the admission Glasgow Coma Scale (GCS) score at the ED, as mild (GCS: 13–15), moderate (GCS: 9–12) or severe (GCS ≤8) [14]. GCS scores were obtained after initial (surgical) resuscitation preferably before sedation and intubation.

Two control groups for both the derivation and the validation study were recruited, one group of healthy controls and a second control group of patients with isolated traumatic orthopedic injuries who were admitted to the ED and the surgical ward. For the derivation study we recruited an extra control group of neurological patients admitted for a central nervous system disease. Including orthopedic trauma patients permitted controlling for factors such as pain and traumatic stress. By including neurological control subjects we aimed to control for aspects of admittance at a hospital ward due to neurological disease.

Exclusion criteria for participants were the following: age below 16 (no upper age limit), previous history of moderate or severe TBI, a history of alcohol and/or drug abuse, previous diagnosis of dementia, and inability to communicate, for example due to aphasia, a tracheostoma or a language barrier. Participants were not included during weekend- or night shifts. However, follow-up testing of included participants was carried out during weekends. Test administrations were also missed if the subject was unable to participate, for instance due to surgery.

The local ethics committee waived the need for review board approval and written informed consent.

### PTA assessment

Assessment of PTA started immediately on presentation to the ED for the mild TBI and orthopedic patients and the day after admittance for the neurological controls. The moderate/severe TBI patients were assessed as soon as possible after regaining consciousness and when they were able to cooperate sufficiently. During derivation and validation a 23-item questionnaire composed of all the individual test items derived from the GOAT [17], MOPTAS [18] and (R-)WPTAS [19,20] scales (Table 1) was administered by the same examiner on a daily basis to the participants until the formal criteria of these tests were met or until discharge or transfer from our hospital. During PTA assessment patients were told the correct answer to a question if the answer was incorrect.

In order to prospectively test anterograde memory and compare the use of pictures against words, patients and controls included in the derivation cohort were randomized using a sealed envelope method for a pictures group (PG) or a words group (WG). For both groups 35 sets, each comprising three memory items, pictures or words, were created as we previously described [23]. At the end of the combined PTA questionnaire the participant was instructed to memorize three items (pictures or words). If free recall was not perfect during the following test (preferably 24 hours after the initial administration and every subsequent 24 hours), the participant had to select the three target pictures or words out of nine distracters. Each time recognition was tested, new sets of distracters were used. In case of errors in recognition, the same three memory items were presented again, and the participant was asked to remember the items until the subsequent test day. The set of target items was changed only in case of flawless recognition. In the (R-)WPTAS, performance on the three-picture test is used to calculate a total test score. In our study, total (R-)WPTAS test scores are based on either the three-picture or the three-word memory test.

Test procedures in the validation study were similar to those described above with three exceptions. First, based on the results of the PTA scale derivation, only words were used as memory items. Second, one extra administration (next to the initial administration and the subsequent assessments each 24 hours) of the composite PTA questionnaire was introduced at one hour after the first administration. Finally, recall and recognition of three words were also tested at five minutes after presentation during the initial questionnaire administration.

### Data analysis

Since no gold standard exists to define PTA, we compared patients and controls, recognizing that not all TBI patients suffered from PTA. The discriminative value of individual test items was determined separately for both the derivation and validation study by calculation of the sensitivity and specificity. The *sensitivity* of a test item was defined as the proportion of TBI patients giving an incorrect answer to that particular item whereas *specificity* referred to the proportion of controls answering the particular test item correctly.

To develop an examiner independent and objective PTA scale, the examiner related items (*name* and *face of examiner*) and the items that cannot objectively be verified for correctness (*last memory preceding* and *first memory following injury*), were excluded from further analysis. Performance of patients and controls was compared using Chi-squared and Fisher’s exact tests: For the orientation items the scores on day one (first administration) and day two were used. We used the score on day two for the memory items. Pearson’s correlation analysis was performed for all items on test day one to determine inter-item correlation. To compose a new PTA scale, items with the highest sensitivity and specificity, taking the inter-item correlation into account, were chosen.

The discriminative value (patients versus controls) of free recall versus recognition of the three-word memory test in the validation sample was determined by computing Receiver Operating Characteristic (ROC) curves. Spearman correlation coefficients were calculated to examine the association between performance on our proposed PTA scale and performance on the GOAT, MOPTAS and (R-)WPTAS scales. Since we used a three-word memory test instead of a three-picture test in this validation cohort the total scores of the MOPTAS and (R-)WPTAS scales were calculated using the three-word recognition scores.

Finally, to compare the performance of our PTA scale with existing scales, we also carried out ROC analyses of the sum scores of all PTA scales to calculate the individual discriminative values (patients versus controls). For all statistical analyses a p <0.05 (two-sided) was considered statistically significant.

## Results

**Figure 1:**
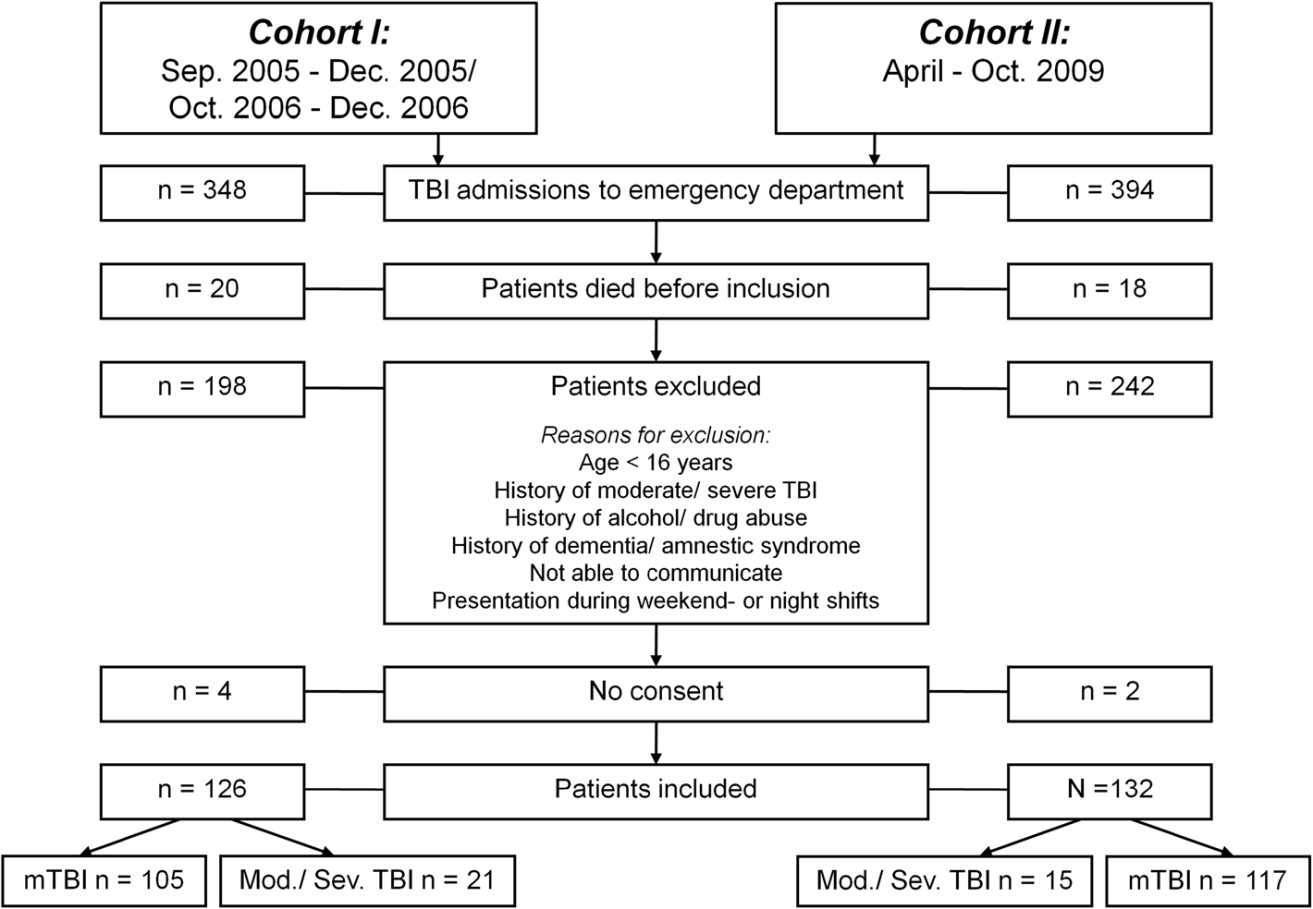
**Inclusion flow diagram of the derivation cohort (Cohort I) and the validation cohort (Cohort II).** Sep., September; Oct., October; Dec., December; TBI, traumatic brain injury; mTBI, mild traumatic brain injury; Mod., moderate; Sev., severe.

Figure 1 shows the inclusion procedure of the TBI patients for both cohorts. In the derivation study 10 healthy, 10 orthopedic (upper or lower leg fracture) and 11 neurological controls (10 patients suffering from a sensorimotor ischemic stroke without aphasia and 1 patient with a benign cerebral tumor) were included. For the validation study we recruited 20 healthy individuals and 10 patients with traumatic orthopedic injuries. The different control groups were collapsed into one single control group per cohort. Table 2 demonstrates the demographic and clinical characteristics of TBI patients and control subjects. Within the derivation study, patients and controls differed considerably in gender and age (Table 2a). For the validation cohort differences were less apparent except for the age difference of nine years between mild TBI patients and controls (Table 2b). The first PTA assessment in the moderate/severe patients included in the derivation cohort was executed at a mean of 17,3 days (standard deviation [sd] 14,9) post-injury, for the validation cohort the mean was 8,6 (sd 10,1) days.

**Table 2:** Participant characteristics

**2a. Derviation cohort**			
	** *Mild TBI* **	** *Moderate/severe TBI* **	** *Control subjects* **
*n*	105	21	31
*Gender (male), n (%)*	64 (61)	18 (86)	12 (39)
*Age, mean (sd)*	46.6 (20.4)	30.1 (16.9)	52.6 (14.2)
*Educational level, n (%)*^*a*^			
- Low	31 (30)	8 (38)	11 (35)
- Intermediate	22 (21)	6 (29)	6 (19)
- High	24 (23)	1 (5)	9 (29)
- missing	28 (27)	6 (29)	5 (16)
*Injury mechanism, n (%)*			
- traffic related	54 (51)	12 (57)	3 (30)
- fall	29 (28)	3 (14)	7 (70)
- violence	-	-	-
- other	22 (21)	6 (29)	-
**2b. Validation cohort**			
*n*	117	15	30
*Gender (male), n (%)*	76 (65)	10 (67)	17 (57)
*Age, mean (sd)*	50.7 (22.3)	39.7 (19.6)	41.4 (18.7)
*Educational level, n (%)*^*a*^			
- Low	28 (24)	1 (7)	8 (27)
- Intermediate	34 (29)	7 (47)	10 (33)
- High	36 (31)	2 (13)	12 (40)
- missing	19 (16)	5 (33)	-
*Years of education, mean (sd)*	13.7 (4.3)	13.3 (3.4)	15.3 (4.8)
*Injury mechanism, n (%)*			
- traffic related	47 (40)	9 (60)	1 (10)
- fall	52 (44)	3 (20)	3 (30)
- violence	6 (5)	-	-
- other	9 (8)	3 (20)	6 (60)
- missing	3 (3)	-	-

Table 3 shows the sensitivity and specificity of individual items administered to the derivation cohort on test days one and two. Moderate/severe TBI patients performed poorer on all items compared to controls except for *name*, *date of birth*, *residence, marital status*, *offspring* and *occupation* (not shown in Table 3). In mild TBI, only *time of day (margin 30 minutes)* on day one and *mode of transport* on days one and two showed a significant difference between patients and controls.

**Table 3:** Discriminative value: Sensitivity and specificity of individual test items on first two days of admission

		**Mild TBI (n = 105/n = 51)**^**a**^		**Moderate/Severe TBI (n = 21/n = 18)**^**a**^		**Controls (n = 31/n = 31)**^**a**^
**Items**		** *Sensitivity (%)* **	** *p Value* **^** *b* **^	** *Sensitivity (%)* **	** *p Value* **^** *b* **^	** *Specificity (%)* **
** *Selected* **						
*Age*	Day 1	6	0.336	19	0.022	100
	Day 2	6	0.292	22	0.016	100
*Name of hospital*	Day 1	11	0.068	50	0.000	100
	Day 2	12	0.412	35	0.011	96
*Time*	Day 1	23	0.002	33	0.001	100
	Day 2	20	0.087	39	0.005	96
*Day of week*	Day 1	11	0.732	33	0.022	93
	Day 2	13	0.088	28	0.008	100
*Month*	Day 1	8	0.198	33	0.001	100
	Day 2	0	-	29	0.006	100
*Mode of transport*	Day 1	19	0.044	48	0.000	97
	Day 2	18	0.025	56	0.000	100
*Recall memory items*	Day 2	46	0.541	71	0.039	62
*Recognition memory items*	Day 2	27	0.273	50	0.013	85
** *Rejected* **						
*City of hospital*	Day 1	4	0.574	24	0.008	100
	Day 2	6	0.547	17	0.062	100
*Period of day*	Day 1	8	0.198	33	0.001	100
	Day 2	10	0.161	33	0.003	100
*Date*	Day 1	25	1.0	62	0.008	77
	Day 2	24	0.122	39	0.021	92
*Year*	Day 1	3	1.0	29	0.013	97
	Day 2	6	1.0	22	0.142	96
*Admission date*	Day 1	29	0.438	71	0.062	62
	Day 2	24	1.0	72	0.001	80

^a^Number of patients between parenthesis: first number on day 1, second number on day 2. ^b^p Value: patients versus controls. TBI, traumatic brain injury.

No significant differences in test performance on the memory items were found between the picture and word groups (data not shown). Both item groups were combined for further analyses. The 24-hours free recall and recognition scores did not differ significantly between mild TBI patients and controls (Table 3), whereas moderate/severe TBI patients performed significantly poorer than controls. The 24-hours free recall of memory items proved to be more difficult than recognition for both patients and controls, as demonstrated by higher sensitivity and lower specificity values.

A correlation was found between *name of hospital* and *city of hospital* (r = 0.61; p <0.0001) and *month*, *day of week*, *time* and *period of day* (0.50 <r <0.56; p <0.0001). Not surprisingly, *date* and *date of admission* showed a correlation in the mild TBI patients (r = 0.67; p <0.0001).

For our PTA scale (Table 4), to be validated in the second part of this study, we selected the items *age*, *name of hospital*, *time*, *day of week*, *month*, *mode of transport* and *recall of three words* after 24 hours based on the following arguments. For the use of a new scale at an ED, we chose items with discriminative value from at least day one in mild TBI patients: *time* and *mode of transport*. Both items showed high specificity (>95%). We selected *age* since it showed significant differences in sensitivity and specificity in moderate/severe TBI patients on at least two consecutive days.

**Table 4:** Proposed new PTA scale

**1.**	**What is your age?**	**…./1**
**2.**	**Where are you now?** (*name hospital*)	**…./1**
**3.**	**What time is it?***(margin 30 minutes)*	**…./1**
**4.**	**Which day of the week do you think it is?**	**…./1**
**5.**	**Which month of the year do you think it is?**	**…./1**
**6.**	**How did you get here?***(mode of transport)*	**…./1**
**On first administration end here and present three words as memory items (procedure see below); on the consecutive administrations (for instance one hour) continue:**		
**7.**	**Can you recall the three words you heard last**	
	**administration?**	**…./3**
*- If less than 3 words are recalled: present the same three words that should have been remembered for this day as memory items.*		
	*While presenting the new words:*	
	**Can you repeat the words I am presenting you?***Immediately after presenting the three words:*	
	**Can you tell me the three words you just have heard?**	
	*If not: present the words once more and have the patient repeat them.*	
** *Score one point for every correct answer to the items 1–6 (maximum 6 points) and 1 point for every word correctly recalled.* **		
		** Sum score …./9**

The items *name of hospital*, *day of week* and *month* were selected because of high specificity and significant differences in test performances between moderate/severe TBI and controls. *Year* demonstrated discriminative value only on day one, hence it was excluded. Items *date* and *admission date* were not selected since specificity was low (≤80%). *Period of day* was not selected because it correlated with *time*. Finally, *city of hospital* was excluded as it correlated with *name of hospital* and proved significant only on day one in the moderate/severe TBI patients.

To test anterograde amnesia, we selected for our new PTA scale words instead of pictures as memory items, as they were not inferior to pictures and might be more practical, especially at ED settings. We preferred recall rather than recognition based on its superior sensitivity.

Table 5 demonstrates the sensitivity and specificity of each individual item of our proposed PTA scale as found in the validation cohort on the three primary administrations (TBI patients and control subjects grouped together). Additionally, data on three-word recognition are given. In the validation cohort specificity of the item *time of day* was lower at the first (86% versus 96%) and 24-hours administration (89% versus 100%). The other items were never failed by the control subjects.

**Table 5:** Sensitivity and specificity of each individual item of the proposed PTA scale

**Item**	** *1* **^** *st* **^** *administration* **		** *one hour after 1* **^** *st* **^** *administration* **		** *24 hours after 1* **^** *st* **^** *administration* **	
	**Sens. (n = 132)**	**Spec. (n = 30)**	**Sens. (n = 21)**	**Spec. (n = 22)**	**Sens. (n = 46)**	**Spec. (n = 19)**
*1. Age*	5	100	0	100	2	100
*2. Name of place*	14	100	5	100	7	100
*3. Mode of transport*	25	100	29	100	15	100
*4. Time*	24	86	29	100	22	89
*5. Day*	13	100	5	100	17	100
*6. Month*	6	100	5	100	7	100
*7. 3-words, recall*	58	77	60	91	77	95
*3-words, recognition*	27	97	25	96	42	100

PTA, post-traumatic amnesia; Sens., sensitivity, %; Spec., specificity, %.

The Area Under the Curve (AUC) of free recall was larger than that of recognition. Consequently, free recall demonstrated better discriminative values: First administration: 0.71 (95% confidence interval [CI]: 0.62-0.79) versus 0.62 (95% CI: 0.52-0.72); second administration: 0.76 (95% CI: 0.61-0.92) versus 0.60 (95% CI: 0.42-0.77); third administration: 0.87 (95% CI: 0.79-0.96) versus 0.71 (95% CI: 0.58-0.84).

Performances on our proposed PTA scale and existing PTA scales showed strong and significant correlations, except for the modest, nevertheless significant, correlations between performances on our PTA scale and the GOAT (Table 6).

**Table 6:** Spearman correlation coefficients demonstrating the relationship between our proposed PTA scale and existing PTA scales

	**Proposed PTA scale**^**a**^		
	** *1* **^** *st* **^** *administration* **	** *2* **^** *nd* **^** *administration (one hour)* **	** *3* **^** *rd* **^** *administration (24 hours)* **
*GOAT*	0.66	0.67	0.59
*MOPTAS*	0.76	0.79	0.81
*WPTAS*	0.74	0.73	0.80
*R-WPTAS*	0.75	0.65	0.76

^a^All correlations were significant at *p* <0.0001. PTA, post-traumatic amnesia; GOAT, Galveston orientation and amnesia test; MOPTAS, modified Oxford PTA scale; (R-)WPTAS, (Revised-)Westmead PTA scale.

Table 7 compares the sensitivity and specificity of the proposed PTA scale with current PTA scales, based on the sum scores of each individual scale (Table 1). Our new PTA scale had largely comparable AUC’s to the existing PTA scales.

**Table 7:** Sensitivity, specificity and AUC’s (ROC analysis) of our proposed PTA scale and existing PTA scales

**PTA Scale**	**1**^**st**^**Administration**				**one hour after 1**^**st**^**administration**				**24 hours after 1**^**st**^**administration**			
	** *Sens.* **	** *Spec.* **	** *AUC* **	** *95% C.I.* **	** *Sens.* **	** *Spec.* **	** *AUC* **	** *95% C.I.* **	** *Sens.* **	** *Spec.* **	** *AUC* **	** *95% C.I.* **
*New PTA scale*	68	77	0.77	0.69 - 0.85	76	91	0.85	0.73 - 0.97	80	95	0.89	0.81 - 0.97
*GOAT*	22	100	0.71	0.63 - 0.80	10	100	0.76	0.61 - 0.91	11	100	0.75	0.63 - 0.86
*MOPTAS*	92	43	0.84	0.77 - 0.90	67	91	0.78	0.64 - 0.93	85	100	0.92	0.86 - 0.99
*WPTAS*	89	43	0.80	0.73 - 0.87	57	91	0.75	0.60 - 0.90	83	100	0.91	0.84 - 0.98
*R-WPTAS*	46	97	0.72	0.64 - 0.80	38	96	0.66	0.50 - 0.83	50	100	0.74	0.63 - 0.86

AUC, area under the curve; ROC, receiver operating characteristic; PTA, post-traumatic amnesia; Sens., Sensitivity,%; Spec., Specificity,%. Based on optimal scores. C.I., confidence interval. GOAT, Galveston orientation and amnesia test; MOPTAS, modified Oxford PTA scale; (R-)WPTAS, Revised-Westmead PTA scale.

## Discussion

We derived and externally validated a new PTA scale consisting of seven objective items including an anterograde memory test of three words. In general, the new scale proved to have equal discriminative capacity compared to existing PTA scales, while being more practical, less time consuming and examiner independent.

An essential feature of ongoing PTA is the inability to store new information. Therefore, anterograde memory testing should be included in a PTA scale [25]. At present, several acknowledged PTA scales, i.e. MOPTAS and (R-)WPTAS, use pictures as memory items. However, the use of words is probably more practical. Moreover, in the derivation cohort the specificity of words as memory items was not inferior to pictures but had a higher sensitivity, confirming earlier findings [22-24]. On the other hand, pictures might still be useful in patients with dysphasia disorders or in case of a language barrier. In line with previous research [23], compared to recognition, free recall of memory items was overall more sensitive and equally specific in diagnosing patients with TBI. Moreover, in the validation cohort, free recall of words showed higher discriminative values (AUC’s: 0.71-0.87) than recognition (AUC’s: 0.60-0.71) on three consecutive administrations. One explanation for this finding may be that after having sustained a TBI recognition of memory items recovers more swiftly than free recall of memory items [22,24,26].

For the external validation of the Nijmegen PTA scale, we recruited a new cohort of participants. Using ROC analysis we studied the discriminative value of our scale across three consecutive administrations in relation to existing PTA scales. During all three test moments our PTA scale showed good discriminative values (AUC’s: 0.77, 0.85 and 0.89) and even the highest value of all scales at the second administration (one hour after initial administration). Although not entirely comparable, the performances of our PTA scale are in agreement with a recent study on the accuracy of the R-WPTAS in the first 24 hours after mild TBI [27]: The specificity of the R-WPTAS was 91%, compared to the 84% maximum specificity of our scale, whereas the sensitivity of the R-WPTAS scale proved 60% and 80% for our proposed PTA scale.

This study has some limitations. Because no gold standard for the assessment of PTA exists, we evaluated test performances of TBI patients against control subjects. The proportion of patients that failed a test item was interpreted as the sensitivity of that particular item. However, as not all TBI patients suffer from PTA [14], the sensitivity of test items appeared relatively low. Specificity was evaluated by administrating the test to control subjects without previous head injury, who consequently did not suffer from PTA. Hence, we think that the specificity of the test items was determined satisfactorily. Furthermore, in the validation study we compared the Nijmegen PTA scale with currently acknowledged PTA scales, which served as surrogate gold standards. In general, we found correlations of more than 0.70 indicating that the level of performance on our PTA scale accurately estimates the cognitive state of the patient. However, no other measures of cognitive functioning were administrated.

In the derivation cohort, some differences in the demographic characteristics existed between patients and controls. The control group consisted of more males, and controls were older than the moderate/severe TBI patients. To our knowledge, no evidence exists that gender effects PTA test performance or PTA emergence. Furthermore, higher age is normally associated with poorer cognitive test performance. Despite their older age, controls were generally able to obtain maximum scores on all test items. In both study cohorts the moderate/severe TBI patients had received less formal education than the mild patients and the controls. This might have influenced the performance of the moderate/severe patients in a negative way, although we think that also most of the lower educated subjects have to be able to answer the questions of the different PTA scales correctly.

Some caution is needed when interpretating the results since the number of moderate/severe TBI patients was restricted possibly reducing the generalizability of our scale to this patient category. And, the number of mild TBI patients tested on consecutive trials decreased which may have increased the sensitivity at later administrations. It is possible that assessing PTA at later stages after injury might have lead to higher sensitivity because better performing patients were already discharged from the hospital. Furthermore, in half of the subjects participating in the derivation study and in all participants of the validation study, words were administered as memory items. However, the MOPTAS and (R-)WPTAS scales normally use a set of pictures to assess a patient’s memory. Substitution of picture recognition scores with word recognition may have influenced the results. Previous studies have shown that picture recognition and recall are less sensitive but equally specific to word recognition and recall [22-24].

We have developed a PTA scale that proved to be accurate in discriminating TBI patients in PTA from control subjects. However, we did not specifically focus our study on the criteria that are required to consider a patient as emerged from PTA. Nevertheless, we consider it reasonable to state that two consecutive maximum test results preclude ongoing PTA.

In this study the inter-rater reliability of our new PTA scale has not been determined. Moreover, we did not study the association of PTA test scores with other TBI (severity) indices - e.g. GCS score, duration of loss of consciousness, and imaging characteristics - and outcome. These additional studies are valuable for further validation of our PTA scale. We also think that an external validation particularly in a larger cohort of moderate/severe TBI patients may be favorable.

PTA assessment may be further improved, especially in patients with MTBI at an ED. We suggest that the diagnostic accuracy of the three-word memory test deserves additional examination, particularly over a time period of 10 to 30 minutes after the first assessment. Moreover, a stricter criterion might be developed on the basis of which a patient can be considered as emerged from PTA. Also, research aimed at a more demanding memory task, for instance a four-item memory test [28], might contribute to the development of more sensitive PTA scales.

## Conclusions

We have developed and externally validated a new examiner independent PTA scale consisting of seven objective items: *age*, *name of hospital*, *time*, *day of week*, *month*, *mode of transport* and *recall ofthree words*. The discriminative capacity of our scale proved comparable to that of several existing PTA scales. Nevertheless, it has the advantage of being more practical and less time consuming. And although more comprehensive validation is recommended, we think that our PTA scale is suitable for TBI patients of all severities.

## Competing interests

The authors declare that they have no competing interests

## Authors’ contributions

BJ: study design, participant inclusion, data acquisition, data analysis and interpretation, manuscript drafting, editing and revision, literature search. JvE: participant inclusion, data acquisition, data analysis and interpretation, manuscript revision, literature search. LV: participant inclusion, data acquisition, data analysis and interpretation, manuscript revision. PD: participant inclusion, data acquisition, data analysis and interpretation, manuscript revision. TA: data analysis and interpretation, manuscript revision. MH: data interpretation, manuscript revision. AvV: participant inclusion, manuscript revision. ME: participant inclusion, manuscript revision. GB: data analysis and interpretation, manuscript revision. PV: study design, data analysis and interpretation, manuscript drafting, editing and revision, takes responsibility for the paper as a whole, guarantees integrity of entire study. All authors read and approved the final manuscript.

## Pre-publication history

The pre-publication history for this paper can be accessed here:

http://www.biomedcentral.com/1471-2377/12/69/prepub
